# Injuries of the Head

**Published:** 1863-09

**Authors:** George K. Amerman

**Affiliations:** Attending-Surgeon to the Chicago City Hospital


### ARTICLE XXIII.

### INJURIES OF THE HEAD.

By Dr. GEORGE K. AMERMAN, Attending-Surgeon to the Chicago City Hospital.

### FRACTURE OF THE SKULL, WITH DEPRESSION; RECOVERY WITHOUT OPERATION.

Case I.—A. H. C., aged 38, in good health, was injured, June 25th, 1863, at about noon. I saw him at 3 P.M., three hours after the accident happened. There were two wounds situated on the head. One, a simple incised scalp wound, located over the parietal eminence of the left side, and of very little consequence. Another, situated over the posterior superior angle of the right parietal, with fracture of the skull and depression. His wound was of an irregular shape, with jagged edges, and must have been produced by some blunt instrument. The portion of skull depressed was about the size of a dime, the depression not exceeding a-quarter of an inch. There were no symptoms of compression,—pulse feeble; extremities and surface of the body cool. Patient talks incoherently; has no knowledge of having received the injury, or that he is at all hurt; cannot remember a single occurrence of the day, not even what happened early in the morning; is constantly inquiring how it happened and wanting to know how he came there. The wound was dressed in the simplest manner, and perfect rest, mentally and physically, enjoined.

June 26th.—Slept some during the night; some pain in the head; pulse 72.

July 1st.—Doing well,—wound suppurating freely; skull bare, and about to exfoilate a small scale of bone; mind clear; not the slightest recollection of anything that took place on the day he was injured. From this time onward he improved daily, and nothing worthy of note occurred.

There are two interesting features connected with the above case,—one physiological, and one practical. The physiological point of interest is, the curious circumstance of the patient loosing all recollection of what took place on the day of the injury. It often happens that a blow on the head will produce complete insensibility, with loss of consciousness, and, if the person recovers, an utter inability to recall a single instance connected with the injury; but we do not think it at all usual for an injury to produce an entire loss of memory as to what occurred hours before the injury was received. The practical point of interest in connection with the case, has reference to the question of trephining, in cases of depressed fracture, in the absence of symptoms of compression.

The remote ill-effects of injuries of the skull, especially fractures, are well known and very much dreaded. Severe and incurable epilepsy has been traced back to some old injury of the head, and even trephining at this late period, resorted to for relief; and yet, it seems to me, the cautious surgeon will hesitate and consider very carefully every circumstance connected with the injury, before he resorts to an operation so important, merely to afford protection from some remote ill-effects which may never occur. If inflammation of the brain and its membranes constitute the principal source of danger, and are to be guarded against in every possible way, then, certainly, the less we interefere by our operations the greater the chance of recovery for our patients.

### CEREBRAL HEMORRHAGE FROM EXTERNAL INJURY. DEATH,—AUTOPSY.

CASE II.—Mrs. McC., aged 40, in ordinary good health, was found, on the morning of the 5th of July, in bed, perfectly insensible, and incapable of being aroused. One or two physicians were called in during the day, who pronounced the case hopeless; and, without any special treatment, she died in the evening. I was called to make the *post mortem* on the 6th.—Weather warm; *post mortem* congestion of dependent portions of the body well marked; arms, front and side of chest covered with black and blue spots; left eye in same condition; scalp ecchymosed in several places over the head,—one large deep ecchymosis just over the left ear; skull uninjured; beneath the dura mater, on the surface of the brain, over the whole of the left side and extending down to the base, there was a thick layer of half-clotted blood, in all, as much as half a pint; surface of the brain normal, except a small spot, which was of a reddish grey color and softened; substance of the brain, at the base, ventricles, and right half were all healthy; abdominal and thoracic organs healthy.

The above case represents, in the very best manner, an instance of fatal compression of the brain, arising from effused blood, the result of an injury. The symptoms, during the lifetime of the patient, were those of compression, and were well marked. The termination, such as might have been, from the symptoms, easily foreseen.

The single point of interest, in connection with the case, is, the occurrence of so great an effusion of blood with so slight an external injury. The ecchymosis of the scalp, which was not extensive or deep, except just above the left ear, was the only external evidence of injury. The pericranium and skull were undisturbed; and no one, from an examination of the scalp, would have regarded the case as a serious one. Treatment would have been useless, even had one been fully aware of the extent of the injury.

PENETRATING WOUND OF THE BRAIN. DEATH,—AUTOPSY.

CASE III.—P. B., aged 35, intemperate, got into difficulty with a neighbor on the evening of the 19th of June, and was struck on the head. The blow was not regarded as serious by his friends, and no surgeon was consulted. He sat up the greater part of the day following, but did not feel altogether right. The next morning, the 21st, he was wandering and talked incoherently. During the forenoon he had several slight convulsions, which increased in severity and became more frequent. In the afternoon, he began to be drowsy and unconscious, and died in the evening.

I was called to make the *post mortem* examination on the 22d, the third day after the injury.—Weather warm; there was one wound situated on the right side of the head, over the parietal eminence, of an irregular shape, and extending through the skull deep into the substance of the brain. It would easily admit the index finger its whole length; scalp ecchymosed about the wound; external table of the skull evenly fractured; internal table jagged and splintered; the wound extended two and a-half inches into the substance of the right hemisphere of the brain, and contained numerous spicula of bone; around this puncture the substance of the brain was softened and of a reddish grey color; all other portions of the brain were healthy; abdominal and thoracic organs healthy.

There is nothing of special interest in the above case, beyond its being a well-marked instance of punctured fracture of the skull, which is rather rare. The fracture of the external table was almost as even and smooth as though the opening had been made with a trephine. The internal table was splintered and some spicula driven into the substance of the brain, but there were no fissures running from the wound. The symptoms preceeding death, delirium, convulsions, and coma were such as might be expected in an injury of this nature.

